# Metabolic and productive responses to heat stress in transition dairy cows: Interactions with calving stage, season, and farm management

**DOI:** 10.1007/s00484-025-03083-3

**Published:** 2026-02-09

**Authors:** E. N. Martínez, C. Castillo, L. Avendaño Reyes, Rodrigo Muiño, L. Díaz-González, J. L. Benedito, J. Hernández

**Affiliations:** 1 Departament of Animal Pathology, Campus Terra-IBADER, University of Santiago de Compostela, Santiago de Compostela, Spain; 2 Institute of Agricultural Sciences, Autonomous University of Baja California, Mexicali, Mexico

**Keywords:** Heat stress, Transition dairy cows, Negative energy balance, Seasonal variation, Metabolic biomarkers

## Abstract

This study investigated the effects of heat stress (HS), calving period, and farm-level management on the metabolic and productive responses of transition dairy cows. Conducted on three commercial farms in northwestern Spain, the study employed a multifactorial design across two seasons (winter and summer) and four peripartum time points. Biochemical parameters, including non-esterified fatty acid (NEFA), β-hydroxybutyrate (BHB), urea, total protein, albumin, glucose, gamma-glutamyltransferase (GGT), and aspartate aminotransferase (ASAT) were analyzed using repeated-measures MANOVA. No significant three-way interactions were found, but several two-way interactions emerged. Notably, NEFA and urea levels varied significantly between farms, while total protein and albumin were influenced by both partum stage and season. Elevated NEFA and BHB concentrations postpartum indicated intensified lipid mobilization and negative energy balance, exacerbated under HS. Reduced albumin and increased urea levels suggested hepatic stress and altered protein metabolism. Farm-specific differences in ASAT during summer highlighted the role of local environmental and management conditions. These findings underscore the complex interplay between physiological stage, environmental stressors, and farm practices. Tailored intervention (such as nutritional adjustments, cooling systems, and precision monitoring) are essential to mitigate the metabolic burden of HS and safeguard cow health and productivity. Future research should explore long-term impacts and adaptive strategies across diverse production systems.

## Introduction

Climate change is increasingly recognised as a major threat to global food systems, and the dairy sector is particularly vulnerable due to the sensitivity of high‑producing animals to environmental stressors. Rising global temperatures and more frequent extreme heat events (now extending into historically temperate regions) are intensifying the incidence of HS in dairy systems that once operated within thermoneutral boundaries (Bernabucci et al. 2010; Polsky and von Keyserlingk 2017). For example, northern Europe and northern Spain now experience thermal stress episodes more regularly, raising concern about long‑term sustainability and animal welfare.

Among the most vulnerable periods for dairy cows is the transition period, which spans approximately three weeks before to three weeks after calving. This phase involves intense physiological, metabolic, and immunological shifts to support calving and the onset of lactation (Sordillo 2016). When temperature and humidity surpass thermoneutral thresholds, typically above 25 °C or a temperature–humidity index (THI) > 68, cows heat‑dissipating mechanisms such as panting, vasodilation, and reduced feed intake (Mader et al. 2006). While these responses maintain body temperature, they can impair milk yield, immune function, and reproduction (Collier et al. 2019; Ramón‑Moragues et al. 2021).

The transition period is already defined by a marked NEB, driven by a sharp increase in nutrient requirements for foetal growth and lactogenesis. HS exacerbates this imbalance by reducing dry matter intake (DMI) and increasing energy expenditure for thermoregulation (Stefanska et al. 2024b). Recent findings show that DMI can decrease by up to 4.13% for every 1-unit increase in the THI Impairing rumen fermentation, acetic acid production, and thus milk fat synthesis (Chen et al. 2024a, b). Rduced intake and elevated physiological load mobilise fat and protein reserves, shown by higher plasma NEFA and BHB biomarkers of intensified lipolysis and hepatic ketogenesis (Rhoads et al. 2013)..

HS impairs hepatic synthesis and induces a metabolic shift towards amino acid deamination for gluconeogenesis (Ma et al. 2019; Sammad et al. 2020). These effects may occur independently of reduced feed intake, indicating that heat load can directly influence hepatic metabolism. Hepatic mitochondrial function is also compromised, with lower ATP production and greater oxidative stress, reducing the energy efficiency needed in early lactation (Sejian et al. 2018; Li et al. 2023b).

Cows under HS exhibit altered behaviour, such as increased standing, disrupted feeding, and reduced rumination (Cook et al. 2007). These adaptations lower nutrient digestibility and feeding efficiency, leading to declines in milk yield and quality, and raising the risk of lameness and digestive disorders (Bernabucci et al. 2010). Global projections estimate that heat stress will cause significant productivity and health losses in livestock, with expected annual damages of up to $39.94 billion by century’s end under high-emission scenarios, heavily impacting dairy production worldwide (Thornton et al. 2022).

Recent studies show that heat stress affects both immediate physiological responses and gene expression linked to metabolism, stress response, and immune regulation in hepatic and mammary tissues (Skibiel et al. 2018). These effects can extend to the next generation, as thermal stress during pregnancy has been linked to altered metabolic and immune phenotypes in calves, indicating potential intergenerational impacts (Halli et al. 2025). The metabolic and molecular patterns of HS, including elevated NEFA, blood urea nitrogen (BUN), liver enzyme activity, reduced protein synthesis, and transcriptional changes, demonstrate a physiological shift favouring survival over productivity. Understanding these mechanisms is crucial for developing strategies that mitigate HS and improve health and performance during the transition period (Sejian et al. 2018). The severity of effects varies with timing relative to parturition and with farm practices such as cooling, nutrition, and housing (Bernabucci et al. 2014), yet integrated studies on these interactions remain scarce. Proposed measures include antioxidant supplements, rumen‑protected nutrients, and precision cooling systems (Abeyta et al. 2023), whose success depends on timely application and adaptation to each farm. A science‑based, context‑specific approach is essential to protect welfare and productivity during the most critical lactation phase.

This study evaluated the effects of heat stress, calving period, and farm-level management on metabolic and productive responses in transition dairy cows, analysing their interactions and identifying critical periods to guide management strategies that improve cow well-being and maintain productivity under heat stress.

## Materials and methods

### Ethical approval

All procedures involving animals were conducted in strict accordance with Spanish legislation (RD 53/2013, legal provision number 1337) and the European Directive 2010/63/EU for the protection of animals used for scientific purposes. The study protocol was approved by the Bioethics Committee of the University of Santiago de Compostela, Spain.

### Experimental design and study location

This study comprised two experiments carried out on three commercial dairy farms (designated as Farm A, Farm B, and Farm C) located in the province of Lugo, Galicia, in northwestern Spain (43°0′N, 7°33′W). The trials were carried out during two distinct seasonal periods: from January to March (winter) and from July to September (summer) of 2023. The farms were situated less than 1 km apart and followed similar management practices.

### Animals and housing

Cows were selected according to the following inclusion criteria: (i) balanced distribution between primiparous and multiparous animals, (ii) body condition score (BCS) between 3.0 and 3.5 at study entry, (iii) absence of clinical disease in the 60 days prior to study entry, and (iv) previous lactation milk yield within ± 5% of the herd average. The distribution by parity and BCS was matched across farms to minimise potential bias in metabolic and productive outcomes.

All farms employed intensive rearing systems. Farm A housed 102 lactating cows, with an average milk yield of 36.86 kg/cow/day, and average milk fat and protein contents of 3.75% and 3.41%, respectively. The barn was oriented north-south, equipped with adjustable dampers for natural ventilation, and exhibited minimal shading at midday due to the shifting position of the sun.

Farm B, located adjacent to Farm A, housed 92 lactating cows with an average daily production of 34.7 kg/cow, cows with an average daily production average milk fat content of 3.76%, and average protein content of 3.39%. Its barn had similar orientation and ventilation settings to Farm A.

Farm C housed 227 lactating cows with an average yield of 33.4 kg/cow/day, and average milk fat and protein contents of 3.80% and 3.36%, respectively. The barn was oriented east-west and had permanent openings on the north and south sides covered with mesh to prevent wildlife entry. Shade patterns shifted from west to east throughout the day.

All farms used a free-stall housing system with individual cubicles of 2.4 m² bedded with straw. Ventilation was provided by roof-mounted fans located above the milking robots, activated when ambient temperature exceeded 24 °C and relative humidity was above 60%. Farm A additionally had a roof-mounted water-sprinkling system using potable water at 14–16 °C, triggered by the same temperature-humidity thresholds.

Each farm utilized robotic milking systems: Farms A and B had two robots in a single barn, whereas Farm C divided its lactating herd into two zones, each with two dedicated robots. All barns featured concrete floors with automated cleaning systems and sand bedding. A total of 152 lactating cows were enrolled in the study: 40 from Farm A, 48 from Farm B, and 64 from Farm C.

### Feeding management

Three lactating cow diets were evaluated, with feed quantities reported as fresh weight alongside their dry matter (DM) contents for clarity. Farm A offered a high-forage diet (69% forage DM) including maize silage (26 kg, 35.7% DM), grass silage (25 kg, 35.7% DM), commercial concentrate (4.5 kg), high-moisture corn (4.0 kg), and barley straw (0.5 kg), providing approximately 21.4 kg DM/day, and energy density 1,67 Mcal/kg DM. Farm B implemented a moderate-forage diet (64% forage DM) with maize silage (27 kg, 39.6% DM), grass silage (13 kg, 39.6% DM), wet grain (2.5 kg), and customized concentrate (5.7 kg), totaling about 19.1 kg DM/day, and energy desnity 1,70 Mcal/kg DM. Farm C’s high-intake, energy-dense diet comprised maize silage (32 kg, 41.7% DM), fresh grass (13 kg, 41.7% DM), barley grain (1.5 kg), robot concentrate (6.2 kg), and a custom lactation mix (6.8 kg), delivering around 24.8 kg DM/day, and energy density 1,72 Mcal/kg DM. Nutrient composition (CP, starch, NFC, NDF, energy) is expressed on a DM basis.

Dry cow diets were also assessed for close-up cows in all three farms: (i) Farm A offered a forage-rich ration (70%) with maize silage (5.0 kg), barley straw (6.0 kg), and concentrate (4.0 kg), supplying 11.9 kg/day of DM (56.7% DM), 13.7% CP, 7.8% starch, 20.7% NFC, 45.1% NDF, and 1.67 Mcal/kg DM; (ii) Farm B included maize silage (11.0 kg), chopped straw (6.0 kg), and dry cow concentrate (3.2 kg), with a total DM intake of 11.6 kg/day (57.6% DM), 13.3% CP, 13.1% starch, 30.3% NFC, 44.6% NDF, and the highest energy density (1.34 Mcal/kg DM); and, (iii) Farm C fed maize silage (10.0 kg), wheat straw (6.9 kg), and custom concentrate (2.8 kg), achieving 11.5 kg/day of DM (58.2% DM), 13.5% CP, 8.7% starch, 19.8% NFC, and the highest NDF (49.7%), with 1.20 Mcal/kg DM.

Nutritional composition was determined using the NDS Professional platform (RUM&N Company, Italy; https://www.rumen.it), developed in collaboration with Cornell University and based on NRC (2001) standards.

### Climate data

Ambient temperature and relative humidity were recorded continuously from January 1 to March 31 and from July 1 to September 30 using a Multi-Use Compact PDF Temperature and Humidity USB Data Logger (Multicomp Pro, Farnell Components SL, Madrid, Spain). The device was mounted 2 m above ground in the separation aisle between lactation zones, out of cows’ reach. It measured air temperature (range − 30 to + 70 °C, ± 0.5 °C accuracy) and relative humidity (± 3% accuracy). Ambient temperature and relative humidity were recorded hourly, and daily mean values were calculated from these hourly measurements for use in statistical analyses.

Measurements were recorded hourly and used to calculate the Temperature–Humidity Index (THI) using the following equation (Mader et al. 2006):

$$\mathrm{THI}\;=\;0.81\;\times\;\mathrm{Ta}\;+\;(\mathrm{RH}\;\div\;100)\;\times\;(\mathrm{Ta}\;-\;14.4)\;+\;46.4$$

In this equation, Ta represents ambient temperature in degrees Celsius (°C), RH represents relative humidity as a percentage (%), and the THI is a dimensionless index commonly used to assess heat load in livestock.

### Blood sampling and biochemical analysis

Each of the 152 cows was sampled at four time points relative to calving: one month prepartum, one-week prepartum, one week postpartum, and one month postpartum. Blood was collected via jugular venipuncture into 10 mL vacutainer tubes (no anticoagulant) between 09:00 and 11:00 a.m. Samples were cooled on ice, centrifuged at 2,500 × g for 10 min at 4 °C within two hours, and serum was aliquoted and stored at − 80 °C.

The following serum parameters were analyzed: (i) Metabolites: glucose (oxidase method), non-esterified fatty acids (NEFA; ACS–ACOD), β-hydroxybutyrate (BHB; enzymatic), total protein (Biuret method), albumin (bromocresol green), creatinine (Jaffé method), and urea (urease method); and (ii) Liver enzymes: aspartate aminotransferase (ASAT) and gamma-glutamyl transferase (GGT).

All assays were performed using a CST-240 autoanalyzer (DIRUI, China), calibrated with Biocal multipoint standards (RAL, Spain), and conformed to quality control protocols.

### Statistical analysis

Data were analyzed using IBM SPSS Statistics v28 (IBM Corp., Armonk, NY). A mixed-design repeated-measures MANOVA was conducted with partum period (four levels: −30 d, − 7 d, + 7 d, + 30 d), season (winter vs. summer), and farm (A, B, C) as fixed factors. Interaction terms (Partum × Season, Partum × Farm, Season × Farm, and Partum × Season × Farm) were included. It is important to note that no significant three-way interaction (partum period × season × farm) was detected for any of the biochemical or productive variables, indicating that the combined effects of partum period and season were consistent across farms (see Table 2).

Compound symmetry was selected as the covariance structure based on the Bayesian Information Criterion (BIC). Mauchly’s test indicated sphericity violations (χ²(5) = 44.20, *P* < 0.001); thus, Greenhouse–Geisser corrections (ε = 0.653) were applied. Normality, homogeneity of variance, and homogeneity of covariance were confirmed via Kolmogorov–Smirnov, Levene’s, and Box’s M tests, respectively (*P* > 0.05).

Estimated marginal means ± standard errors (SEM) were reported. Statistical significance was declared at *P* ≤ 0.05. When significant interactions were detected, Bonferroni-adjusted pairwise comparisons were performed. Individual animals nested within farms were modelled as a random effect to account for repeated measurements.

## Results

Table 1 summarizes the statistical significance (P-values) of the main effects (partum period (PP), sampling season (SS), and farm (F)) as well as their two-way interactions (PP × SS, PP × F, and SS × F) for each biochemical and productive parameter. Significant effects are highlighted in bold (*P* ≤ 0.05), allowing for the identification of key physiological and environmental influences on cow metabolism and performance.

**Table 1:** P-values for the main effects and interactions of PP, SS, and F on biochemical and productive parameters

P value							
Item	PP	SS	F	PP x SS interaction	PP x F interaction	SS x F interaction	PPxSSxF interaction
Tot Protein	**<,001**	**,048**	,279	**,013**	**,015**	,701	,190
Abumin	,052	,731	,182	**<,001**	,105	,870	,216
GGT	**<,001**	,448	,245	,422	,284	,588	,132
ASAT	**,002**	**,296**	,228	,916	,263	**,036**	,174
Urea	**,001**	**,005**	,228	,104	**<,001**	,237	,222
Glucose	**<,001**	,238	,629	,932	,088	,123	,792
NEFA	**<,001**	,781	,721	,272	**<,001**	,156	,143
BHB	**<,001**	,474	,935	,967	,735	,851	,085
THI	**,050**	**<,001**	**,041**	**<,001**	**,019**	**,003**	,609

Partum period (PP): 1mbe: 1 month before partum; 1wbe: 1 week before partum; 1 wpos: 1 week post partum; 1mpos: 1 month post partum. Samling Season (SS): W: Winter; S: Summer. Farms (F): A: Farm A; B:Farm B; C: Farm C: C. SEM: Standard Error of the Mean. ^4^The P*C*T interaction was not significant (P>0.05) for any response variable

The PP × SS (Table 2) interaction was significant for total protein and albumin. One month before partum, cows sampled in summer exhibited higher serum total protein concentrations (7.99 ± 0.05 g/dL) compared with those sampled in winter (6.98 ± 0.05 g/dL; *P* = 0.010; Table 2, line 1). For serum albumin, a seasonal effect was detected one month postpartum, with higher values in winter (3.16 ± 0.01 g/dL) than in summer (2.87 ± 0.01 g/dL; *P* = 0.050; Table 2, line 2). The interaction effects that reached statistical significance (*P* < 0.05), including relevant P-values, means ± SEM for each group, and key time points or conditions where differences were observed, are detailed in Table 2. Different superscripts denote significant differences among groups.

**Table 2:** (a) Statistically significant interaction effects between PP × SS on metabolic and production parameters (b) Statistically significant interaction effects between PP × F on metabolic and production parameters (c) Statistically significant interaction effects interaction effects between SS × F on metabolic and production parameters

**Parameter**	**P-value**	**Mean ± SEM**	**Condition of Significant Difference**
Total Protein	,010	W: 6.98±0.05 a S: 7.99±0.05 b	1mbe
Albumin	,050	W: 3.16 ±0.01 a S: 2.87 ±0.01 b	1mpos
THI	,001	W: 59.47 ±0.20 a S:70.77 ± 0.20 b	1mbe
	,001	W: 58.03 ±0.22 a S: 74. 77 ±0.14 b	1wbe
	,001	W: 60.55 ±0.16 a S: 74. 15± 0.13 b	1wpos
	,001	W:61.99 ± 0.15 a S: 74.33 ± 0.13 b	1mpos
**Parameter**	**P-value**	**Mean ± SEM**	**Condition of Significant Difference**
Total Protein	,010	Farm A: 7.27 ±0.04 a Farm B: 7.12 ±0.04 a Farm C: 6.71±0.3 b	1wbe
Urea	,001	Farm A: 24.71 ±0.39 a Farm B: 29.29 ±0.32 a Farm C: 18.60±00.27 b	1mbe
	,004	Farm A: 20.94 ±0.37 a Farm B: 26.46 ±0.30 b Farm C: 20.24±0.25 c	1wbe
	,014	Farm A: 30.10 ±0.40 a Farm B: 23.29 ±0.32 b Farm C: 27.01±0.27 c	1mpos
NEFA	,029	Farm A: 0.15 ±0.00 a Farm B: 0.12 ±0.01 b Farm C: 0.19±0.00 c	1mbe
	,010	Farm A: 0.14 ±0.01 a Farm B: 0.16 ±0.01 a Farm C: 0.37 ±0.00 b	1wbe
	,034	Farm A: 0.57 ±0.01 a Farm B: 0.56 ±0.01 a Farm C: 0.37±0.01 b	1wpos
	,001	Farm A: 0.42 ±0.01 a Farm B: 0.38 ±0.17 a Farm C: 0.22±0.00 b	1mpos
THI	,003	Farm A: 69,88 ±0.51 a Farm B: 64.54 ±0.3 b Farm C: 67.45 ± 0.24 ab	1wpos
THI	,008	Farm A: 69,17 ±0.49 a Farm B: 66.43 ± 0.30 ab Farm C: 64.31 ± 0.24 b	1mpos
**Parameter**	**P-value**	**Mean ± SEM**	**Condition of Significant Difference**
ASAT	,036	Farm A: 97.72 ±1.51 a Farm B: 76.04 ±1.32 b Farm C: 69.86 ± 1.24 b	Summer
THI	,003	Farm A: 63,81 ±0.24 a Farm B: 58,34 ±0.15 b Farm C: 57,23±0.14 b	Winter

Partum period (PP): 1mbe: 1 month before partum; 1wbe: 1 week before partum; 1 wpos: 1 week post partum; 1mpos: 1 month post partum. Season (SS): W: Winter; S: Summer. Farms (F): A SEM: Standard Error of the Mean. Differences are expressed as means ± SEM, with superscript letters indicating significant differences between conditions

Partum period (PP): 1mbe: 1 month before partum; 1wbe: 1 week before partum; 1 wpos: 1 week post partum; 1mpos: 1 month post partum. SEM: Standard Error of the Mean

This table summarizes the significant interactions (P ≤ 0.05) between the physiological stage of the animals and the farm of origin, reflecting potential management or environmental differences across locations. Values are presented as means ± SEM, with different superscript letters denoting statistically significant differences

Partum period (PP): 1mbe: 1 month before partum; 1wbe: 1 week before partum; 1 wpos: 1 week post partum; 1mpos: 1 month post partum. Season (SS): W: Winter; S: Summer. Farms (F): A: Farm A; B: Farm B; C: Farm C. SEM: Standard Error of the Mean

This table shows the significant interactions (P ≤ 0.05) between season and farm, highlighting how environmental and management conditions may differentially affect metabolic responses. Data are expressed as means ± SEM, and superscript letters indicate significant differences among farms within each season

A significant interaction between PP × F (Table 3) was observed for serum total protein, serum urea, and serum NEFA. One week before partum, serum total protein concentrations were higher in farms A and B (7.27 ± 0.04 and 7.12 ± 0.04 g/dL, respectively) than in farm C (6.71 ± 0.03 g/dL; *P* = 0.010; Table 2, line 7). Serum urea concentrations varied notably across farms at multiple time points: one month prepartum, farm B (29.29 ± 0.32 mg/dL) and farm A (24.71 ± 0.39 mg/dL) had higher values than farm C (18.60 ± 0.27 mg/dL; *P* < 0.001; Table 2, line 8); one week prepartum, farm B (26.46 ± 0.30 mg/dL) exceeded farm A (20.94 ± 0.37 mg/dL) and farm C (20.24 ± 0.25 mg/dL; *P* = 0.004; Table 2, line 9); and one month postpartum, farm A (30.10 ± 0.40 mg/dL) and farm C (27.01 ± 0.27 mg/dL) had higher values than farm B (23.29 ± 0.32 mg/dL; *P* = 0.014; Table 2, line 10).

**Table 3:** Statistically significant interaction effects between PP × F on metabolic and production parameters

Parameter	P-value	Mean ± SEM	Condition of Significant Difference
Total Protein	,010	Farm A: 7.27 ±0.04 a Farm B: 7.12 ±0.04 a Farm C: 6.71±0.3 b	1wbe
Urea	,001	Farm A: 24.71 ±0.39 a Farm B: 29.29 ±0.32 a Farm C: 18.60±00.27 b	1mbe
	,004	Farm A: 20.94 ±0.37 a Farm B: 26.46 ±0.30 b Farm C: 20.24±0.25 c	1wbe
	,014	Farm A: 30.10 ±0.40 a Farm B: 23.29 ±0.32 b Farm C: 27.01±0.27 c	1mpos
NEFA	,029	Farm A: 0.15 ±0.00 a Farm B: 0.12 ±0.01 b Farm C: 0.19±0.00 c	1mbe
	,010	Farm A: 0.14 ±0.01 a Farm B: 0.16 ±0.01 a Farm C: 0.37 ±0.00 b	1wbe
	,034	Farm A: 0.57 ±0.01 a Farm B: 0.56 ±0.01 a Farm C: 0.37±0.01 b	1wpos
	,001	Farm A: 0.42 ±0.01 a Farm B: 0.38 ±0.17 a Farm C: 0.22±0.00 b	1mpos
THI	,003	Farm A: 69,88 ±0.51 a Farm B: 64.54 ±0.3 b Farm C: 67.45 ± 0.24 ab	1wpos
THI	,008	Farm A: 69,17 ±0.49 a Farm B: 66.43 ± 0.30 ab Farm C: 64.31 ± 0.24 b	1mpos

Partum period (PP): 1mbe: 1 month before partum; 1wbe: 1 week before partum; 1 wpos: 1 week post partum; 1mpos: 1 month post partum. SEM: Standard Error of the Mean

This table summarizes the significant interactions (P ≤ 0.05) between the physiological stage of the animals and the farm of origin, reflecting potential management or environmental differences across locations. Values are presented as means ± SEM, with different superscript letters denoting statistically significant differences

Serum NEFA concentrations also differed among farms. One month before partum, farm C (0.19 ± 0.00 mmol/L) showed higher values than farms A (0.15 ± 0.00 mmol/L) and B (0.12 ± 0.01 mmol/L; *P* = 0.029; Table 2, line 11). One week before partum, farm C (0.37 ± 0.00 mmol/L) remained higher than A (0.14 ± 0.01 mmol/L) and B (0.16 ± 0.01 mmol/L; *P* = 0.010; Table 2, line 12). In contrast, one week postpartum, serum NEFA concentrations were higher in farms A and B (0.57 ± 0.01 and 0.56 ± 0.01 mmol/L, respectively) compared with farm C (0.37 ± 0.01 mmol/L; *P* = 0.034; Table 2, line 13), and this pattern persisted one month postpartum (0.42 ± 0.01 and 0.38 ± 0.17 mmol/L vs. 0.22 ± 0.00 mmol/L; *P* = 0.001; Table 2, line 14).

**Fig. 1:**
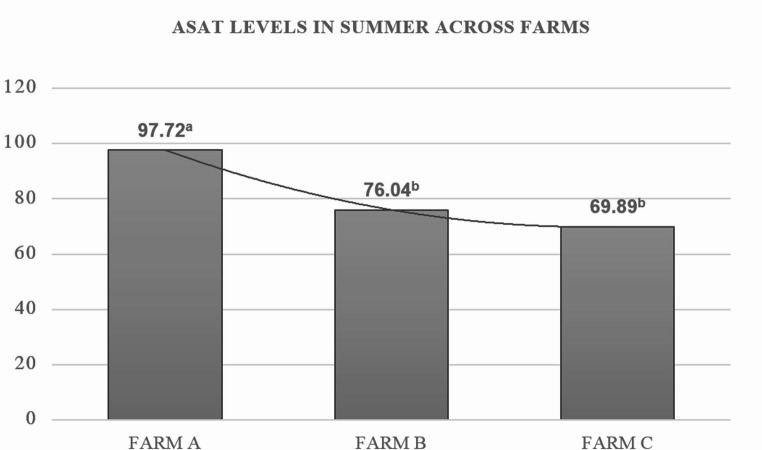
Statistically significant interaction effects between season and farm (SS × F) on metabolic and production parameters (P-Value 0,036)

A significant SS × F interaction was detected for serum ASAT activity (Fig. 1). During summer, ASAT activity was greatest in farm A (97.72 ± 1.51 U/L), compared with farm B (76.04 ± 1.32 U/L) and farm C (69.86 ± 1.24 U/L; *P* = 0.036; Table 2, line 18).

**Fig. 2:**
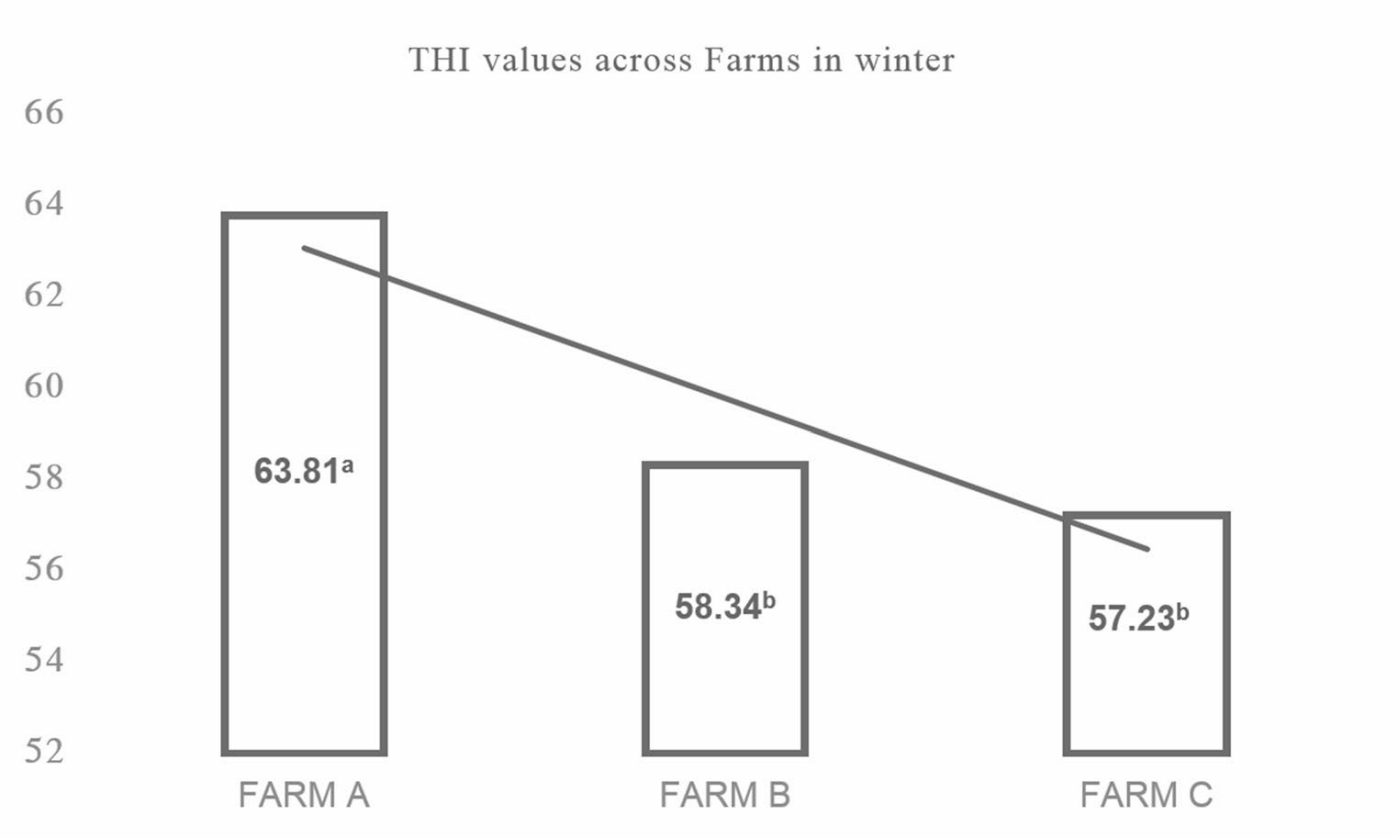
Statistically significant interaction effects between SS × F on metabolic and production parameters (P-Value 0,003)

In addition, the THI, a key indicator of heat stress, exhibited significant variation across several factors (Fig. 2). THI was significantly affected by the partum period (*P* = 0.050) (Table 4), Season (*P* < 0.001) (Table 5), and farm (*P* = 0.041) (Table 6), with notable two-way interactions observed for PP × SS (*P* < 0.001), PP × F (*P* = 0.019), and SS × F (*P* = 0.003; Table 1, row ‘THI’). As shown in Table 5, THI values were markedly higher in summer (72.68) than in winter (60.00), and also varied among farms, being highest in Farm A (67.97) compared to Farms B (65.04) and C (66.00) (Table 6). More specifically, THI values were significantly higher in summer than in winter at all time points: one month before partum (70.77 ± 0.20 vs. 59.47 ± 0.20; *P* < 0.001), one week before partum (74.77 ± 0.14 vs. 58.03 ± 0.22; *P* < 0.001), one week postpartum (74.15 ± 0.13 vs. 60.55 ± 0.16; *P* < 0.001), and one month postpartum (74.33 ± 0.13 vs. 61.99 ± 0.15; *P* < 0.001). Farm-specific differences were also evident: one week postpartum, THI was highest in farm A (69.88 ± 0.51), followed by farm C (67.45 ± 0.24) and farm B (64.54 ± 0.30; *P* = 0.003); one month postpartum, farm A again showed the highest THI (69.17 ± 0.49), followed by farm B (66.43 ± 0.30) and farm C (64.31 ± 0.24; *P* = 0.008). During winter, THI remained highest in farm A (63.81 ± 0.24), compared to farm B (58.34 ± 0.15) and farm C (57.23 ± 0.14; *P* = 0.003). These findings highlight the influence of environmental and management conditions on thermal stress, which may have downstream effects on metabolic and productive responses during the transition period.

**Table 4:** Estimated means values of biochemical and productive parameters according to partum period

Item	Partum period				
	1mbe	1wbe	1wpos	1mpos	SEM
Total Protein(g/dl)	**7,48**^a^	**6,89**^b^	**6,66**^b^	**7,21**^a^	0,17
ALBUMIN(g/dl)	3,03	2,94	2,91	3,01	0,06
GGT (UI/l)	**25,81**^a^	**20,04**^b^	**21,20**^b^	**26,53**^a^	0,17
ASAT (UI/L)	**66,86**^a^	**71,33**^a^	**104,29**^b^	**72,09**^a^	1,07
UREA (mg/dl)	**24,20**^ab^	**22,55**^a^	**26,58**^b^	**26,80**^b^	0,22
Glucosa (mg/dl)	**85,72**^a^	**79,75**^ab^	**71,13**^b^	**73,27**^b^	0,41
NEFA (mmol/L)	**0,158**^a^	**0,228**^a^	**0,503**^b^	**0,344**^c^	0,04
BHB (mmol/L)	**0,52**^a^	**0,51**^a^	**0,74**^b^	**1,10**^c^	0,01
THI	65,07	66,30	67,33	66,66	0,08

Values are presented for four time points relative to parturition: 1 month before (1mbe), 1 week before (1wbe), 1 week postpartum (1wpos), and 1 month postpartum (1mpos). Different superscripts within rows indicate statistically significant differences (P < 0.05). SEM, standard error of the mean

**Table 5:** Estimated means values of biochemical and productive parameters according to season

Item	Season		
	W	S	SEM
Total Protein(g/dl)	**6,86**^a^	**7,27**^b^	0,20
Albumin(g/dl)	2,99	2,96	0,06
GGT (UI/l)	22,61	24,18	0,24
ASAT (UI/L)	**75,58**^a^	**81,24**^b^	0,42
UREA (mg/dl)	**23,15**^a^	**26,92**^b^	0,12
Glucose (mg/dl)	79,80	75,12	0,23
NEFA (mmol/L)	0,30	0,31	0,03
BHB (mmol/L)	0,74	0,69	0,06
THI	60,00^b^	72,68^b^	0,08

Values are presented two sampling seasons (winter [W] and summer [S]). Different superscripts within rows indicate statistically significant differences (P < 0.05)

*SS* Season, S*EM* standard error of the mean

**Table 6:** Estimated means values of biochemical and productive parameters according to farm

Item	Farms (F)			
	A	B	C	SEM
Total Protein(g/dl)	7,11	7,22	6,86	0,25
Albumin(g/dl)	3,05	2,97	2,90	0,08
GGT (UI/l)	25,99	22,15	22,02	0,22
ASAT (UI/L)	84,41	77,85	72,92	0,61
UREA (mg/dl)	25,69	25,89	23,52	0,27
Glucose (mg/dl)	79,74	77,54	75,09	0,42
NEFA (mmol/L)	0,325	0,308	0,292	0,01
BHB (mmol/L)	0,709	0,737	0,715	0,01
THI	67,97^a^	65,04^b^	66,00^b^	0,09

Values are presented for three farms (A, B, and C). Different superscripts within rows indicate statistically significant differences (P < 0.05)

*F* farm, *SEM* standard error of the mean

Regarding main effects, and excluding parameters involved in significant two- or three-way interactions, the partum period (Table 4) significantly influenced serum GGT activity, serum glucose, and serum BHB concentrations. Specifically, serum glucose levels declined around parturition, while BHB concentrations increased progressively from prepartum to one month postpartum (0.52 to 1.10 mmol/L), consistent with intensified ketogenesis during negative energy balance. GGT activity also varied across the peripartum period, suggesting hepatic stress or altered liver function during the transition phase. These findings are consistent with the physiological adaptations expected in dairy cows undergoing metabolic stress during calving.

The estimated means (± SEM) of all biochemical and productive parameters across the four partum periods (Table 4), two Seasons (Table 5), and three farms are presented in Table 6. These tables provide a comprehensive overview of the main effects, allowing for the identification of temporal and environmental trends in the metabolic profiles of transition cows.

## Discussion

This study provides novel insights into the intricate relationships between HS, physiological stage, and farm-level management in shaping the metabolic and productive responses of transition dairy cows. The results of the present study serve to reinforce the multifactorial nature of HS impacts, as evidenced by significant two-way interactions among partum period, season, and farm for key metabolic and productive indicators. These results highlight the importance of context-specific management strategies to mitigate the adverse effects of HS, particularly during the biologically vulnerable transition period.

Following preceding research, the present data substantiate the notion that HS during the transition period intensifies the NEB intrinsic to this stage by diminishing DMI and augmenting maintenance energy requirements through thermoregulatory mechanisms (Bernabucci et al. 2014; Polsky and von Keyserlingk 2017). Recent meta-analytic evidence indicates that DMI can decline by 0.85 to 1.23 kg/day for each unit increase in THI above 68 (Hammami et al. 2013; Chen et al. 2024a, b), which in turn compromises rumen fermentation, acetic acid production, and de novo milk fat synthesis. This metabolic disruption is supported by earlier reports demonstrating reduced ruminal volatile fatty acid production and altered microbial populations under HS conditions (Wheelock et al. 2010; Zeng, et al., 2022). Consequently, bovines experience intensified adipose and muscle catabolism, as reflected in elevated serum NEFA and BHB, reliable biomarkers of lipid mobilization and hepatic ketogenesis (Gross et al. 2011; Rhoads et al. 2013).

The findings further demonstrate the occurrence of evident disruptions in protein metabolism and hepatic function. This is evidenced by increased BUN levels, along with reduced serum albumin and total protein concentrations. These alterations suggest impaired hepatic protein synthesis and increased amino acid catabolism to support gluconeogenesis, a pattern previously associated with HS in metabolomic and proteomic studies (Ma et al. 2019; Sammad et al. 2020). Notably, the observed metabolic changes appear to be independent of feed intake, thereby providing support for the hypothesis that heat load exerts a direct influence on hepatic mitochondrial function, resulting in the induction of oxidative stress (Li et al. 2023a, b; Marquez-Acevedo, et al., 2023). Interestingly, lower serum urea concentrations observed in Farm C may be explained by a reduced proportion of rumen degradable protein (RDP) in the diet, which has been shown to decrease serum urea nitrogen levels due to improved nitrogen utilization efficiency (Tacoma et al. 2017).In addition to lipid-related biomarkers, alterations in glucose and GGT concentrations further support the presence of metabolic and hepatic stress during the transition period. The observed decline in glucose levels postpartum may reflect increased peripheral glucose uptake for lactogenesis, coupled with reduced hepatic gluconeogenesis under heat stress conditions (Wheelock et al. 2010; Zeng, et al., 2022). Meanwhile, elevated GGT activity (particularly during the periparturient period) may indicate hepatic oxidative stress or cholestatic responses, as previously reported in cows exposed to thermal load (Sejian et al. 2018; Sammad et al. 2020). These findings reinforce the hypothesis that heat stress not only disrupts energy metabolism but also impairs liver function through both direct and indirect mechanisms.

Proteomic analyses have demonstrated that HS exerts a regulatory effect on mitochondrial respiratory chain proteins and heat shock proteins in hepatic tissue, thereby compromising energy production and cellular homeostasis (Sejian et al. 2018; Skibiel et al. 2018). These mitochondrial dysfunctions are consistent with the findings of Collier et al. (2019), who emphasised the role of mitochondrial bioenergetics disruption in HS pathophysiology.

Beyond the biochemical parameters, HS instigates behavioural adaptations such as an increase in standing time, a decrease in lying and rumination, and an alteration in feeding frequency. Consequently, these adaptations decrease the digestibility of nutrients and exacerbate metabolic stress (Fritz 2021; Ramón-Moragues et al. 2021). Such behavioural shifts have been demonstrated to have a detrimental effect on immune function, thus increasing susceptibility to mastitis, lameness, and gastrointestinal disorders (West 2003). Recent studies also suggest that HS-induced changes in the neuroendocrine system may exacerbate inflammatory responses, further compromising health. As demonstrated by St-Pierre et al. (2003) and Key et al. (2014), HS have been shown to result in significant reductions in milk production, reproductive efficiency, and longevity. This, in turn, has considerable negative impacts on the dairy industry.

The significant interactions identified between partum period and season for total protein and albumin, and between partum period and farm for NEFA and urea, reveal the intertwined effects of environmental and nutritional factors on cow metabolism. Seasonal fluctuations in total protein levels before parturition, and decreases in albumin postpartum, particularly during summer months, may be indicative of the cumulative impacts of subclinical inflammation and oxidative stress (Stefanska, et al., 2024). The observed variability among farms in NEFA and urea concentrations is likely to be indicative of differences in nutritional strategies (e.g. energy density, protected amino acids), cooling infrastructure, and the implementation of monitoring protocols such as rumen sensors and panting scores (Correa-Calderón et al. 2022; Fabris et al. 2019). The interaction between the season of sampling and the farm on which the samples were collected further emphasises the importance of environmental stressors that are specific to the farm. Evidence suggests that elevated ASAT levels in Farm A during the summer months are indicative of inadequate thermal regulation or insufficient welfare management. This highlights the need for customised mitigation strategies tailored to each farm’s infrastructure, microclimate, and herd characteristics (Bernabucci et al. 2014; Sejian et al. 2018). Additionally, differences in dietary formulation and nutritional strategies across farms may have contributed to the observed metabolic variability. Variations in energy density, protein degradability, and the inclusion of feed additives such as rumen-protected amino acids or antioxidants could influence parameters such as NEFA, urea, and total protein (Fabris et al. 2019; Correa-Calderón et al. 2022). These nutritional factors, in combination with environmental stressors, likely shaped the physiological responses observed in each farm. Although serum albumin levels appeared lower in summer compared to winter one month postpartum, this difference was observed within the interaction between partum period and sampling season (PP × SS). However, no significant effect was detected when comparing seasons as a main factor (Table 4), where values were similar (2.99 vs. 2.96 g/dL) and lacked statistical superscripts. Therefore, the seasonal variation in albumin should be interpreted cautiously and attributed to interaction effects rather than a standalone seasonal influence (Stefanska, et al., 2024). While the discussion highlights lower serum urea concentrations in Farm C, this pattern was most evident in the interaction effects (PP × F), particularly at specific time points such as one month before and after partum (Table 1, lines 8–10). In contrast, the main effect of farm (Table 5) showed more comparable values across farms (25.69, 25.89, and 23.52 mg/dL), with no statistical superscripts indicating significance. Thus, the observed differences in urea are better explained by farm-specific interactions rather than by farm as an isolated factor (Tacoma et al. 2017).

Significantly, recent omics studies have revealed that HS has long-lasting molecular consequences. Transcriptomic analyses have shown the upregulation of genes involved in inflammation and stress (e.g., HSP70, NF-κB), and the downregulation of genes related to lipid metabolism in hepatic and mammary tissues (Skibiel et al. 2018; Li et al. 2023a, b). Epigenetic modifications associated with prenatal heat exposure have been linked to persistent metabolic alterations and immune dysregulation in offspring, raising concerns about transgenerational impacts (Halli et al. 2025; Zeng, et al., 2022). Notably, analogous transgenerational effects have also been documented in rodent models exposed to thermal stress during gestation, thereby underscoring the broader biological significance. In line with these findings, the THI emerged as a critical environmental variable influencing the metabolic responses of transition cows. The present study revealed significant effects of THI across partum period, sampling season, and farm, as well as their two-way interactions (PP × SS, PP × F, SS × F), underscoring the multifactorial nature of thermal stress. THI values were consistently higher in summer than in winter across all physiological stages, with the most pronounced differences observed one week before partum (74.77 ± 0.14 vs. 58.03 ± 0.22) and one month postpartum (74.33 ± 0.13 vs. 61.99 ± 0.15). Farm-specific differences were also evident, particularly during the postpartum period, with Farm A exhibiting the highest THI values both in summer and winter. These variations likely reflect differences in microclimatic conditions, ventilation systems, and heat abatement strategies (Bernabucci et al. 2014; Sejian et al. 2018). The significant interaction between THI and physiological stage suggests that cows in late gestation and early lactation are particularly vulnerable to heat load, which may exacerbate the metabolic shifts associated with negative energy balance (Polsky and von Keyserlingk 2017;(Zeng, et al., 2022). These findings reinforce the importance of incorporating THI monitoring into herd management protocols, particularly during the transition period, to anticipate and mitigate the physiological burden imposed by heat stress (Stefanska et al. 2024b).

It is evident that mitigation strategies, including antioxidant supplementation, rumen-protected amino acids, and evaporative cooling, have demonstrated efficacy in reducing the metabolic burden of HS (dos Santos et al. 2016; Abeyta et al. 2023). However, the effectiveness of these strategies is contingent on their timely application and adaptation to the specific conditions of the local farm. The observed inter-farm variability underscores the significance of precision livestock farming tools, including real-time THI monitoring, automated feeding systems, and wearable sensors, in informing decision-making and optimising welfare (Stefanska et al. 2024b; Papakonstantinou et al. 2024). Furthermore, the incorporation of genetic selection for thermotolerance, utilising genomic markers such as slick hair and heat shock protein polymorphisms, has been identified as a potential long-term strategy for enhancing resistance (Cheruiyot et al. 2022). Recent advances in gene editing and epigenetic modulation present potential future avenues to enhance heat resilience (Worku et al. 2023).

Notwithstanding the strengths of this study, including its multifactorial design, real-farm conditions, and robust statistical modeling, certain limitations should be acknowledged. The geographical scope was restricted to three commercial farms within a single climatic region, which may limit the generalizability of the findings to broader contexts. Future research should aim to include a wider range of geographic and climatic conditions, as well as incorporate additional outcome measures such as reproductive performance and immunological status. Moreover, evaluating the long-term effects of heat stress and mitigation strategies on metabolic homeostasis and productivity is warranted to better understand transgenerational impacts.

## Conclusions

This study shows that heat stress during the transition period in dairy cows causes significant metabolic disruptions, with effects varying by calving stage, season, and farm management. Key metabolic markers, NEFA, urea, and total protein, were notably altered under heat stress, indicating increased negative energy balance and physiological strain. Significant two-way interactions were found for example, NEFA and urea levels varied between farms, while total protein and albumin were affected by both partum stage and season. These results highlight that both environmental and management factors shape the metabolic response to heat stress. Therefore, tailored interventions (such as nutritional adjustments, cooling systems, and precision monitoring) are essential to mitigate these impacts and protect cow health and productivity in the face of rising climate challenges. Future research should explore the long-term effectiveness of these strategies across diverse production systems and climatic conditions, with particular attention to their impact on metabolic homeostasis and health outcomes in subsequent lactations.

## Data Availability

The data utilized in this article will be made available upon reasonable request from interested parties.
